# Genetic Structure in the Seabuckthorn Carpenter Moth (*Holcocerus hippophaecolus*) in China: The Role of Outbreak Events, Geographical and Host Factors

**DOI:** 10.1371/journal.pone.0030544

**Published:** 2012-01-24

**Authors:** Jing Tao, Min Chen, Shi-Xiang Zong, You-Qing Luo

**Affiliations:** 1 Beijing Forestry University, Beijing, People's Republic of China; 2 Silviculture and Conservation of Ministry of Education, Beijing Forestry University, Beijing, China; Instituto de Higiene e Medicina Tropical, Portugal

## Abstract

Understanding factors responsible for structuring genetic diversity is of fundamental importance in evolutionary biology. The seabuckthorn carpenter moth (*Holcocerus hippophaecolus* Hua) is a native species throughout the north of China and is considered the main threat to seabuckthorn, *Hippophae rhamnoides* L. We assessed the influence of outbreaks, environmental factors and host species in shaping the genetic variation and structure of *H. hippophaecolus* by using Amplified Fragment Length Polymorphism (AFLP) markers. We rejected the hypothesis that outbreak-associated genetic divergence exist, as evidenced by genetic clusters containing a combination of populations from historical outbreak areas, as well as non-outbreak areas. Although a small number of markers (4 of 933 loci) were identified as candidates under selection in response to population densities. *H. hippophaecolus* also did not follow an isolation-by-distance pattern. We rejected the hypothesis that outbreak and drought events were driving the genetic structure of *H. hippophaecolus*. Rather, the genetic structure appears to be influenced by various confounding bio-geographical factors. There were detectable genetic differences between *H. hippophaecolus* occupying different host trees from within the same geographic location. Host-associated genetic divergence should be confirmed by further investigation.

## Introduction

Pests with fluctuating population size are of major concern for forest security. Knowledge of a pest's population dynamics and associated influential factors is crucial for forest management. Habitat, weather, natural enemies and heritable traits are considered to play roles in insect population dynamics [1]. Despite many studies, the factors involved in the origin of insect outbreaks remain poorly understood. Multiple explanations have been proposed including: escape from natural enemies [2]–[5], favorable weather [6], changes in host quality and quantity [7]–[9], and genetic variation of pests [10]–[12].

The seabuckthorn carpenter moth, *Holcocerus hippophaecolus* Hua (Lepidoptera: Cossidae) is the main pest of seabuckthorn, *Hippophae rhamnoides* L. (Elaeagnaceae). It usually occurs on seabuckthorn, but can also occur on *Ulmus pumila* L. (Urticales: Ulmaceae) as well as a couple of species of Rosaceae [13]. The larvae seriously obstruct water transportation of seabuckthorn by boring into the trunk and roots. It has one generation every 3–4 years and larval stages occupy most of its life history. It is widely distributed throughout its hosts' range, with most damage being caused to trees more than 5 years old. The adult females have limited dispersal and lay their eggs in masses on nearby plants where the larvae feed gregariously. Berryman [14] has demonstrated that pests with low dispersal properties have short, intense, restricted outbreaks whereas those with high vagility have long, extended outbreaks. Consistent with the former pattern, the seabuckthorn carpenter moth has limited dispersal ability and exhibits short but intense outbreaks that are geographically restricted [15]. Zhou reported that outbreaks of *H. hippophaecolus* can lead to more than 70% mortality of seabuckthorn in plantations in the Inner Mongolia Autonomous Region [16]. Limited mobility appears to play a role in the spatial restriction of the seabuckthorn carpenter moth. The outbreaks usually continue for one or two years before pest numbers decline [15], [17].

Seabuckthorn is native to western and northern China, the northern Himalayas and northwestern Europe, through to central Asia and the Altai Mountains [18]. It is a native in 11 provinces (autonomous region, municipalities) in China, with less than 500 thousand hectares of natural forest in the 1950's [19]. Because of seabuckthorn's nitrogen-fixing symbionts, this plant serves to enrich and protect soils [18]. It has been promoted widely in western and northern China to prevent soil erosion and desertification. There are now 2,000,000 ha of seabuckthorn throughout 22 provinces in China, two-thirds of which are monoculture plantations. *H. hippophaecolus* was firstly reported as a pest of seabuckthorn in 1990 [20]. Today *H. hippophaecolus* is considered to be the main threat to seabuckthorn in China. It infests 133,000 ha of seabuckthorn and killed 67,000 ha during the 1990's. Most of the outbreak events occurred in Seabuckthorn monoculture plantations [16]. Prior to the spread of *H. rhamnoides* plantations in western and northern China, no outbreak events of *H. hippophaecolus* had been recorded. *H. rhamnoides* was introduced as a novel plant to Jianping County (Liaoning Province) in the 1950's. Prior to that, *H. hippophaecolus* mainly fed on *U. pumila* [16], [19]. Jianping has been cultivating seabuckthorn widely since the 1970's and used to have the largest area of seabuckthorn plantation. However, by 2001, the forests were heavily disturbed by the seabuckthorn carpenter moth.

Molecular markers are widely used for insect population genetic research and are a useful tool to study population structure. Correlation with ecological factors, to identify causes of the observed genetic structuring, is often possible using these techniques [21], [22]. The Amplified Fragment Length Polymorphism (AFLP) technique generates a large number of fragments that are distributed throughout the genome, without requiring background knowledge of the genome [23]. In the present study, the AFLP technique was used to examine population genetic patterns of *H. hippophaecolus* to address the questions below.

### Are genetic patterns a factor in causing outbreaks?

Natural selection, favoring certain genotypes at low densities and others at high densities, may contribute to the regulation of animal numbers [10]. Many studies have demonstrated that the genetic patterns may play a role in population dynamics. For example, changes of allelic frequencies of different loci have been shown to correlate with population fluctuations in *Microtrous ochgaster* [24]. Simchuk et al [25] suggested that esterase (*Est-4*) and protease (*Pts-4*) loci in *Tortrix viridana* L. were directly related to its population dynamics.Mormon crickets (*Anabrus simplex*) were found to consist of genetically distinct clusters that correspond with gregarious (outbreak) populations and solitary (non-outbreak) populations, respectively [11]. These genetic clades provided evidence that the differences of propensities to outbreak are likely due to genetic polymorphism. The seabuckthorn carpenter moth is a very destructive pest, with outbreaks reported in many areas within its range (outbreak areas), but it exists at low densities in other parts of its range (non-outbreak areas). *H. hippophaecolus* populations may therefore consist of genetically distinct clusters with different propensities for outbreak. Here we describe the genetic variation of *H. hippophaecolus* populations from 10 areas across its range with contrasting historical patterns of outbreak events [15]–[17], [26], [27] and assess whether the observed genetic patterns can be explained by outbreak history.

### Do geographical distance, outbreaks and drought affect genetic population structure?

Identifying the factors responsible for structuring genetic diversity is important for a better understanding of the insect's evolutionary history. Such fundamental studies help to infer ecological characteristics that are crucial for establishing management strategies. Geographical distance, outbreaks, and drought were considered in this study. First, in the case of species with restricted dispersal abilities, we expect to observe a positive correlation between the genetic and geographical distance. Moreover, in the historical outbreak regions, *H. hippophaecolus* undergoes outbreak events that are followed by population decline when food plants become unavailable. Such population fluctuations may have large effects on genetic diversity within populations. Furthermore, non-genetic studies indicate recent outbreak events were related to a lack of rainfall [16]. Heavy periodic rainfalls could restrict *H. hippophaecolus* movement and genetic exchange within populations. Therefore, we might also expect lower genetic differentiation within populations during drought.

### Does host-associated diversity exist in a common locality?

Herbivores and their host plants maintain an intimate relationship in feeding, oviposition, mate finding and predator avoidance. Distribution, availability, longevity and chemistry of host plants are major factors that affect the genetic differentiation of herbivorous populations [28]. Seabuckthorn was a novel host plant for insects following its introduction into Jianping. Herbivore populations can suffer from disruptive selection following shifts to novel host plants [29]. How did they adapt to the changes? Is there detectable genetic diversity among *H. hippophaecolus* populations feeding on different host trees in a common location? To answer these questions, we sampled larvae from sympatric populations of four host trees, sebuckthorn, *U. pumila*, *Prunus armeniaca* L, (Rosales: Rosaceae), *Pyrus pyrifolia* (Burman) Nakai (Rosales: Rosaceae), in Jianping.

## Materials and Methods

### Sample collection and DNA extraction

**Figure 1:**
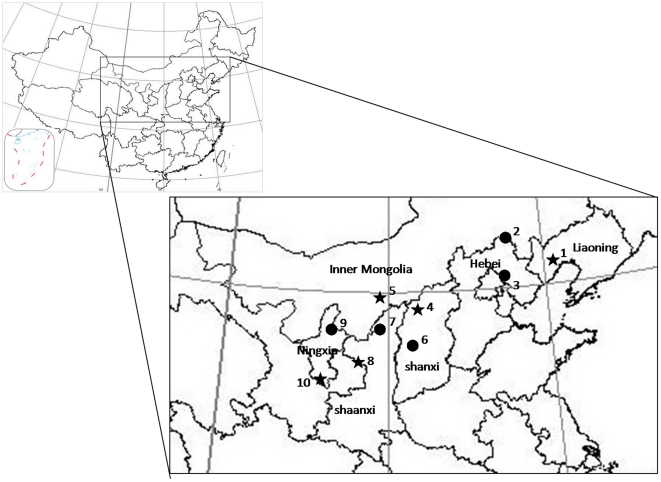
Locations of 10 *H. hippophaecolus* sites with known outbreak (star) or non-outbreak (circle) patterns. Outbreak patterns based on Luo et al. [16], population 1; Wang [26], poulation 4; Zhou [15], population 5; You [27], population 7; Zong et al. [17] population 10; Zong, personal communication, population 2,3,6,7,9. Identities and locations of populations see Table 1.

Individuals (n = 217) were collected from 10 locations across the carpenter moth range during the summer of 2008 (Table 1) by directly sampling under the bark of infested trees and byusing light and pheromone traps. Sampling locations represented two contrasting patterns of historical outbreak events, based on a literature survey and unpublished data (J. Zong, personal communication) (Figure 1). Populations from some areas have experienced outbreaks while in others population densities have been consistently low. In Jianping, a further 24 insects were collected from different hosts (*U. pumila* (JPY, n = 7), *Prunus armeniaca* (JPX, n = 8), *Pyrus pyrifolia* (JPL, n = 9)). Individuals were transported alive to the laboratory, and then kept at −80°C. Prior to DNA extraction, insects were washed in 80% ethanol. Total genomic DNA was isolated using the SDS-method of Zhang and Hewitt [30]. After extraction, DNA was dissolved in TE buffer and stored at −20°C until further use.

**Table 1:** Geographical location, average annual rainfall, and hosts for populations of *H. hippophaecolus*.

No.	Location	Coordinates	Average annual rainfall/mm	Population indentifier	Host plant	Sample Size
1	Liaoning,Jianping	119.71E/41.84N	478.37	JPS	*H.rhamnoides*	26
				JPY	*U. pumil*	7
				JPX	*P. armeniaca*	8
				JPL	*P. pyrifolia*	9
2	Inner mongolia,Linxi	118.23E/43.61N	378.98	LX	*H.rhamnoides*	16
3	Hebei,Fengning	116.61E/41.21N	535.16	FN	*H.rhamnoides*	25
4	Shanxi,Youyu	112.39E/39.96N	377.24	YY	*H.rhamnoides*	26
5	Inner mongolia,Dongsheng	111.25E/39.87N	410.52	DS	*H.rhamnoides*	15
6	Shanxi,Wuzhai	111.87E/38.88N	431.54	WZ	*H.rhamnoides*	18
7	Shanxi,Yulin	109.76E/38.28N	397.43	YL	*H.rhamnoides*	19
8	Shanxi,Wuqi	108.26E/36.90N	534.83	WQ	*H.rhamnoides*	29
9	Nixia,Yanchi	107.48E/37.89N	290.24	YC	*H.rhamnoides*	22
10	Nixia,Pengyang	106.50E/35.82N	498.67	PY	*H.rhamnoides*	21

### AFLP protocol

Amplified fragment length polymorphism (AFLP) analysis was used to assess genetic diversity among sampled populations of *H. hippophaecolus*. The AFLP procedure followed Vos et al. [23] with minor modifications. Genomic DNA was digested with *Eco*RI and *Mse*I restriction enzymes (New England Biolabs) and double stranded adapters were ligated to the sticky ends of the fragments. After 4 h incubation at 37°C, each sample was diluted 1∶9 with H_2_O and a two-step amplification strategy was used. Pre-selective amplification was performed for 3 min at 94°C, then 30 cycles of 30 s at 94°C, 30 s at 56°C and 1 min 72°C. A 20-µl Pre-selective amplification PCR mixture consisted of 30 mM MgCl_2_, 4.5 mM dNTP, 0.6 U Taq DNA polymerase, 30 ng *Eco*RI-C and *Mse*I-A primer. In the selective amplification, we used the following nine primer combinations selected from 100 tested combinations [31]: *Eco*RI-AAC/*Mse*I-CAA, *Eco*RI-AAC/*Mse*I-CAC, *Eco*RI-AAC/*Mse*I-CCT, *Eco*RI-AAC/*Mse*I-CTT, *Eco*RI-AAG/*Mse*I-CCA, *Eco*RI-AAG/*Mse*I-CTG, *Eco*RI-CA/*Mse*I-CAA, *Eco*RI-CA/*Mse*I-CAC, *Eco*RI-CA/*Mse*I-CCT. The *Eco*RI primers were labeled with IRD-700. Selective amplification was performed with the following touchdown thermal profile: 3 min at 94°C; 12 touchdown cycles at 94°C for 30 s, 65°C for 30 s (decreasing the temperature by 0.7°C per cycle), and 72°C for 60 s; 30 cycles at 94°C for 30 s, 56°C for 30 s, 72°C for 1 min; 5 min at 72°C. The 10 µl PCR mixture contained 15 MgCl_2_, 1.5 ng *Mse*I and *Eco*RI primer, 2 mM dNTP), 2 µl diluted (1∶9) Pre-amplified DNA. All PCRs were conducted on a GeneAmp PCR System 9700 (USA Applied Biosystems).

Amplification products were separated on 6% polyacrylamide gels for 2.5 h on a LI-COR 4300 DNA Analyzer (LI-COR Biosciences, USA), using LI-COR 50–700 bp (Labeled with IRD-700) as a size standard. Fragments from 100–700 bp in size were scored as present (1) or absent (0) using SAGA MX (LI-COR Biosciences, USA), and exported for data analysis.

A blank control was carried out along with each set of DNA extractions and PCR amplifications to monitor any possible cross contamination. Poor-quality DNA samples that did not amplify were excluded from further analysis.

### Data analysis

#### Genetic variation and structure of *H. hippophaecolus* populations

The diversity of geographic populations was assessed by estimating the percentage of polymorphic loci (%P) and Nei's heterozygosity. Percentage of polymorphic loci estimates were based on 99% criteria and heterozygosity estimates were made using the software TFPGA [32].

The genetic structure was examined by an analysis of molecular variance (AMOVA) performed by the software ARLEQUIN 3.1 [33]. This method was used to partition the genotypic variance among and within populations. Two separate analyses were performed to test the hypotheses of genetic structure attributable to variation: among individuals across the different localities feeding on *H. rhamnoides* and among individuals across different host plants in Jianping. An additional analysis of individuals feeding on *H. rhamnoides* compared to the group combining three other host plants in Jianping was also performed. Genetic differentiation coefficients between populations (both geographic and host-associated) were calculated as *F_ST_*, with 95% confidence intervals (CI) obtained by bootstrapping 1000 replicates over loci. The TFPGA software was also applied to calculate Nei's genetic distance (D) [34]. Neighbor-joining (NJ) trees were constructed based on D using MEGA4 [35].

#### Identification of candidate outbreak loci under selection

Outlier loci were identified using the Dfdist approach [36], [37] in Mcheza program [38] (available at http://popgen.eu/soft/mcheza/). Allele frequencies are estimated in Dfdist based on Zhivotovsky's [39] Bayesian approach.

Because of our particular interest in outbreak-associated divergence, the Dfdist was run for two groups of populations (outbreaking population vs non-outbreaking population). A total of 50000 realizations were performed and maximum allowable allele frequency was 0.99. We chose the 0.995 confidence interval and set the significance level at 99%. The Benjamini and Hochberg false discovery rate (FDR) correction method was used to correct for the occurrence of false positives in loci identified as under selection [40]. Loci with significant *P*-values at FDR threshold of 50% were identified using the Benjamini and Hochberg method.

#### Testing outbreaks and environmental factors driving genetic structure

The following analysis tested outbreaks and environmental factors that potentially influenced genetic population structure. The effect of geographical distance was assessed using linear map distances between *H. hippophaecolus* populations. Secondly, outbreak patterns were scored with 1 indicating populations from areas where outbreaks had occurred and 0 representing populations in non-outbreaking areas. Finally, an index for the “degree of drought,” represented by the average annual rainfall collected over 50 years was obtained (1955–2007, China meteorological data sharing service system http://cdc.cma.gov.cn/). Mantel tests were conducted with the software TFPGA to test the correlation between Euclidean distances for all the factors and genetic distances.

The general linear models (GLM) method was also used to test the effect of outbreak and drought on the genetic differentiation between populations. In this analysis the factor “drought” was defined as locations with less than 400 mm average annual rainfall. Values of 1 were used for drought locations (YY, YL, YC, LX) and 0 for other locations (PY, JP, WQ, DS, WZ, FN). The outbreak factor was standardized, as previously, for an outbreak area of 1 and a non-outbreak area of 0. We performed a GLM analysis of the heterozygosity with outbreak and drought as fixed factors. A *P*-value of <0.05 was used to indicate statistical significance. GLM was implemented using SPSS 16.0.

## Results

### Genetic variation and structure of *H. hippophaecolus* populations

The nine primer combinations produced a total of 933 bands. The global 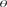 among the 10 sites was 0.2106 (95% CI 0.1981–0.2230). Nei's heterozygosity for each geographical population was moderate and ranged from 0.1505∼0.2042 (Table 2).

**Table 2:** Percentage of polymorphic loci (%P) and Nei's heteozygosity of *H. hippophaecolus* populations.

	Population indentifier									
	JP	LX	FN	YY	DS	WZ	YL	WQ	YC	PY
**%Polymorphic loci (p)**	72.56	51.23	77.60	58.41	74.49	68.27	70.85	66.34	56.38	61.74
**Heterozygosity (H)**	0.1854	0.1529	0.1702	0.1495	0.2042	0.1505	0.1872	0.1749	0.1604	0.1679

AMOVA conducted on AFLP markers confirmed the presence of moderate genetic differentiation showing that 22.54% of total variability was due to the variation among geographic populations (*F_ST_* = 0.2254, *P*<0.0001) (Table 3). The pair-wise comparisons between populations were characterized by values of F*_ST_* ranging from 0.0424–0.3663 (Table 3). Most of the populations showed highly significant differences (*P*<0.0001) with the exception of the YY and LX populations (*P* = 0.0182). This result indicates that most of the 10 sampled populations represent differentiated populations.

**Table 3:** Analysis of molecular variance (AMOVA) of *H. hippophaecolus* populations.

Source of variation	d.f.	SS	Percentage of variation (%)	P
*Geographical grouping*				
Among localities	9	5831.247	22.54	<0.0001
Individuals within localities	206	18395.396	77.46	<0.0001
*Host-plant grouping*				
Among host plants in Jianping	3	1537.343	31.73	<0.0001
Individuals within host plants in Jianping	30	3895.897	68.27	<0.0001
Among two host groups in Jianping	1	1249.317	34.82	<0.0001
Individuals within groups in Jianping	48	4183.923	65.18	<0.0001

**Figure 2:**
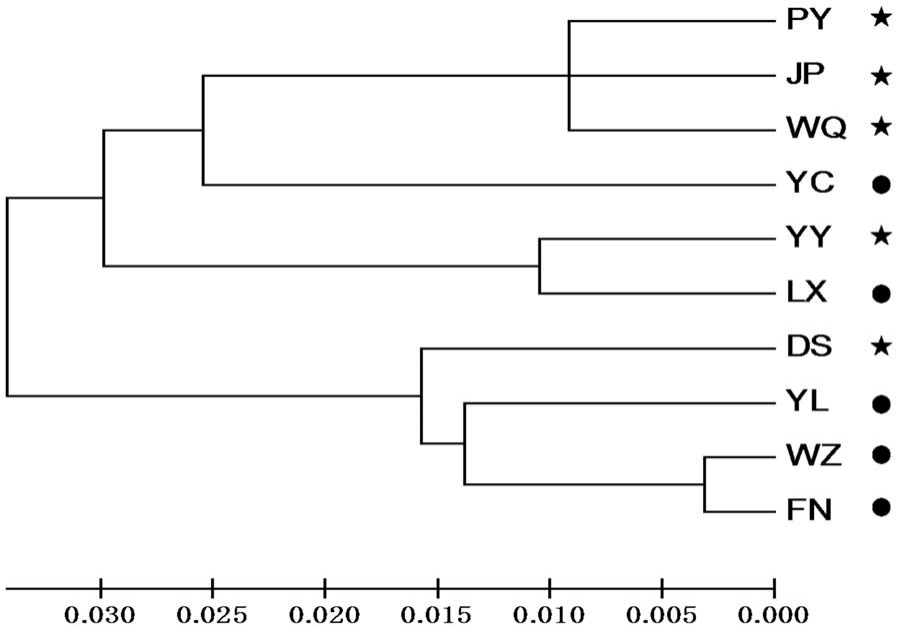
Neighbor-joining phenogram of Nei's genetic distances between *H. hippophaecolus* populations at different collection sites. Site names and outbreak patterns correspond to those in figure 1. Populations are depicted as outbreaking (star) or non-outbreaking (circle).

The Neighbor Joining phenogram (Figure 2) indicates that the clusters comprised populations with a mixture of outbreak patterns. For instance, populations from Dongsheng and Youyu were in two distinct NJ genetic clusters, although they have the same intensity of outbreak events.

**Figure 3:**
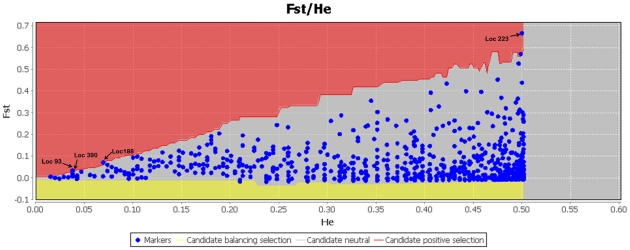
Result of Dfdist analyses in Mcheza. F_ST_ against heterozygosity plot is provided. Loci were identified as significant, corresponding to a 99% critical frequency and 99.5% confidence interval. Red and yellow lines represent 0.995th and 0.005th quantiles of the conditional distribution obtained from Dfdist simulations respectively. Each blue dot indicates AFLP marker; dots in gray areas represent neutral loci falling below the 0.995 quantile's broad line, whereas outlier loci in red areas are pointed out by arrows and accompanied by the locus number.

Examination of the AFLP data using Dfdist in Mcheza sought to determine whether there was evidence of any highly differentiated loci. F_ST_ is plotted against heterozygosity in Figure 3. The outbreak and non-outbreak population comparison performed with Dfist resulted in four markers out of 993 (loci 93, 188, 223, 390) showing more differentiation than expected at the 99.5% confidence level. All these loci were detected as potential positive outliers at the 50% FDR threshold (Figure 3).

### Testing outbreaks and drought as factors driving *H. hippophaecolus* genetic structure

The Mantel test based on the 10 localities gave an *r* value of 0.0554 (*P* = 0.3460, for 10000 randomizations), indicating no correlation between geographic and genetic differences. The Nei's genetic distances between populations were not significantly correlated to outbreak differences in the Mantel test (r = 0.2516, *P* = 0.0740). The interaction between Euclidean distances for average annual rainfall and genetic distances was also not significant (Mantel test r = 0.1271, *P* = 0.2070). GLM analysis showed that the factors of outbreak and drought, and their interaction, did not have a significant effect on heterozygosity (F_1,10_ = 0.053, *P* = 0.826, F_1,10_ = 1.329, *P* = 0.293 and F_1,10_ = 2.904, *P* = 0.139 respectively).

### Host-associated diversity

The host plant was found to have a larger effect on the genetic structure among populations than geographic location. The global 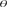 value among different hosts was 0.2785 (95% CI 0.2548–0.3024), higher than the value among 10 sites (0.2106). AMOVA with ARLEQUIN found greater variation among populations in host-plant groupings (31.73%) than populations in geographical groupings (22.54%) (Table 3). Pairwise *F_ST_* statistics between JPS and each other location population ranged from 0.0856 to 0.2978 (Table 4), while the genetic divergences were all highly significant 0.3510∼0.3773 in the host-associated analysis (Table 5).

**Table 4:** Nei's genetic distance and F*_ST_* value between all geographic combinations.

	PY	JP	YY	WQ	DS	YL	WZ	YC	LX	FN
PY	—	0.0902	0.2409	0.0683	0.1589	0.2179	0.2929	0.2779	0.2601	0.2298
JP	0.0191	—	0.2682	0.0856	0.1969	0.2204	0.2978	0.2754	0.2737	0.2467
YY	0.0465	0.0587	—	0.2654	0.1115	0.2938	0.3663	0.3477	0.1047	0.2715
WQ	0.0170	0.0196	0.0583	—	0.1813	0.2048	0.2914	0.2404	0.2856.	0.2454
DS	0.0401	0.0442	0.0423	0.0468	—	0.1550	0.1828	0.2511	0.1089	0.0986
YL	0.0530	0.0559	0.0888	0.0527	0.0362	—	0.1129	0.1642	0.2520	0.1298
WZ	0.0816	0.0780	0.1247	0.0869	0.0375	0.0254	—	0.2998	0.3131	0.0424
YC	0.0538	0.0541	0.0774	0.0447	0.0617	0.0437	0.0871	—	0.3387	0.2819*
LX	0.0456	0.0578	0.0209	0.0606	0.0343	0.0678	0.0944	0.0731	—	0.2126
FN	0.0669	0.0652	0.0969	0.0740	0.0207	0.0298	0.0062	0.0827	0.0705	—

Nei's genetic distances are below the diagonal. F*_ST_* value and their significance level are above the diagonal. Significance level of associated F*_ST_* value are shown as:

*0.01<P<0.05, unmarked mean P<0.0001.

**Table 5:** Pairwise comparisons of genetic divergence estimates (*F_ST_*) between all host plants combinations.

	JPY	JPL	JPX	JPS
JPY	0.00000			
JPL	0.07590*	0.00000		
JPX	0.05271*	0.11808**	0.00000	
JPS	0.36080***	0.37730***	0.35109***	0.00000

Significance level of associated F*_ST_* value are shown as:

*0 01<P<0.05;

**0.001<P<0.01;

***P<0.0001.

In Jianping, individuals feeding on *H. rhamnoides* had a great separation from individuals feeding on other host plants. When combined individuals feeding on *U. pumila*, *P. armeniaca* and *P. pyrifolia* as a group, compared to individuals feeding on *H. rhamnoide*, the variation among two groups rose up to 34.82% by AMOVA with ARLEQUIN. Pairwise comparisons of *F_ST_* values between all host plant combinations further supported the pattern of genetic structure. *F_ST_* values were much greater in comparisons between the *H. rhamnoides* feeders (0.3510–0.3773) and each other host-plant feeders (0.0527–0.1180) (Table 5). *H. rhamnoides* feeders showed strongly significant differences (*P*<0.0001) with the moth on other host plants (Table 5).

## Discussion

### Genetic patterns associated with outbreak events of *H. hippophaecolus*

Genetic clustering did not support distinct outbreak-associated genetic clades in *H. hippophaecolus*. NJ genetic population clusters contained a combination of populations from historical outbreak areas as well as non-outbreak areas (Figure 2). The outbreak effect may have been difficult to detect among different geographical populations due to various confounding biogeographical factors that also shape genetic structure in *H. hippophaecolus*. In addition, one cannot exclude the possibility that the outbreak and non-outbreak patterns are associated with a single genotype, but depend on the expression of different phenotypes in different environments.

Indeed, our results support the notion that outbreak events were likely to be endemic population changes from latent to epidemic rather than being due to insects with an outbreak-associated genotype spreading to outbreak areas. This conclusion is also consistent with the poor dispersal ability of *H. hippophaecolus*, which has been observed in non-genetic studies. Zong et al. [17] indicated female moths usually choose a nearby tree for mating after emergence, and that the body weight of fertilized female moths is too heavy for long-distance migration. Males are not attracted to sex pheromone traps located too far away from the infested forests (<100 m) [13]. Furthermore, adults live for only several days, which limits the degree of dispersal. Young seedlings (1–2 years) and seeds of seabuckthorn are often used for its introduction. However, *H. hippophaecolus* only harms seabuckthorn plants that are more than 5 years old. Therefore, *H. hippophaecolus* would not be dispersed long-distances by artificial movement of host plants.

We rejected the hypothesis of genetic difference associated with outbreaks in the seabuckthorn carpenter moth. However, habitat, weather, natural enemies are also considered as main factors affecting insect population dynamics. An outbreak occurs when the physiological state of the plant permits a herbivore phenotype with a high reproductive capacity to become dominant. Agricultural and forest monocultures consisting of extensive plantings of hosts with narrow genetic variability are havens for pest outbreaks [1]. Seabuckthorn monoculture plantations are optimal sites for survival of *H. hippophaecolus*, especially for those that were introduced as an exotic species growing under unfavorable conditions [41]. Weather and climatic conditions significantly affect population fluctuation. Unusual weather is known to have strong effects on the dynamics of insect populations [42], [43]. Several authors associated the initiation of outbreaks in Jianping and Dongsheng with consecutive dry years before outbreak [15], [19,]. Seabuckthorn plantations have plenty of nutrients, relatively few natural enemies and are highly vulnerable to drought or human disturbance, which may explain why outbreak events happen there.

The outlier analysis revealed that although differentiation for the majority of markers did not significantly deviate from neutral expectations, a small number of markers (n = 4) were identified as outlier loci. The false discovery rate test supported the conclusion that all identified outlier loci are under selection. These results do not allow us to reject the hypothesis that specific genome regions or genes are associated with outbreak events. Having indentified outlier loci in *H. hippophaecolus*, it will be necessary to try to find candidate genes that could correspond to these AFLP markers. Then we can characterize these genes with functional genomics analyses.

### Factors influencing the population structure of *H. hippophaecolus*

We found evidence of limited gene flow among samples collected from 10 locations. The poor correlation between genetic and geographical distances is an unexpected result given the previous assumption that populations are isolated-by-distance.

These results suggest that multiple factors other than simple geographic distance are influencing the genetic composition of populations. The Mantel analysis failed to support the idea that outbreak and drought are pertinent factors underlying genetic structure in *H. hippophaecolus*. Compared to other observations in insects, Nei's heterozygosity values for each population are moderate [44], [45], [46]. We found similar genetic diversity within outbreak and non-outbreak populations of the seabuckthorn carpenter moth. Similarly, GLM analysis showed that outbreaks had no significant effects on heterozygosity. Genetic diversity within outbreak populations was not strongly affected by increases in population size during outbreak periods. This result could be consistent with the theoretical prediction that long-term fluctuating populations correspond to the harmonic mean size over time, and should thus be closer in size to that during the remission period than during an outbreak [47], [48]. We rejected the hypothesis that drought populations have lower genetic variation. GLM analysis showed that drought had no significant effects on heterozygosity.

Comparative studies of population structure in phytophagous insects show that genetic structure is mostly determined by the ability of the species to disperse [49]. The probability of successful dispersal is largely determined by habitat availability [50], [51]. Louy et al. [52] have shown in experiments with three skipper species that dispersal ability and habitat availability determine the genetic structure of species. Whether a habitat is available for phytophagous insects strongly depends on the existence of host trees. Several forest insects have pronounced geographical structure that follows the distribution of their host tree species [46], [53].

Louy et al. [52] suggest that limited habitat availability in combination with low dispersal capacity result in independent genetic structure with relative high genetic differentiations and low gene flow among populations. *H. hippophaecolus* is a specialist, which only feeds on a few plants (*U. pumila*, *P.armeniaca*, *P.pyrifolia*). Moreover, most of the principal host in China, *H. rhamnoides*, is in single species plantations which are suffering from human interference. Could this independent population structure of *H. hippophaecolus* be explained by low dispersal capacity and habitat fragmentation? Combining habitat landscape and population genetic analysis might answer this question in the future.

### The role of the plant

Host races are genetically differentiated sympatric populations of parasites that use different hosts and between which there is limited gene flow [54]. Our analyses uncovered very high F*_ST_* values (0.3510–0.3773) between JPS and other non-seabuckthorn populations. It is indicated that *H. rhamnoides* constitutes a barrier to gene flow between *H. hippophaecolus* populations from other host plants in Jianping. *H. hippophaecolus* feeding on *H. rhamnoides* in Jianping are more genetically differentiated than those from other hosts in sympatric rather than other geographically distant populations of seabuckthorn. Host races might therefore exist in seabuckthorn and other host plant used by *H. hippophaecolus*. Factors favoring host race formation include correlations between host choice and mate choice. Although host fidelity and assortative mating has not been fully explored in *H. hippophaecolus*, tests using both artificial and natural methods suggest female host preferences may exist. Adult emergence from the seabuckthorn roots confirmed oviposition preference on *H. rhamnoides*, rather than on *U. pumila* and *P. armeniaca* [55].

Seabuckthorn was an endemic perennial, sporadically growing in Inner Mongolia, Shanxi and areas of Liaoning province before it was widely promoted. The timing of host shifting of *H. hippophaecolus* in Jianping is likely due to the introduction of *H. rhamnoides*. However, how did host shifting occur in *H. hippophaecolus* in Jianping? When did host-associated genetic divergence initially occur in *H. hippophaecolus*? Data from many host utilization systems gave rise to a possible scenario that host shifts occur as a result of host plant's increased abundance and availability as a potential resource following human-mediated plant community changes [56], [57]. If this is the case, our data suggests a local host shift and genetic differentiation of *H. hippophaecolus* following the introduction of seabuckthorn in Jianping. Though a rapid range expansion of *H. hippophaecolus* following human-mediated changes is possible, it does seem unlikely given the wide extent of genetic divergence observed during such a brief time. This scenario was also rejected by Sword et al in the *Hesperotettix viridis* host utilization system [58]. Another possibility is a genetic divergence of moth between *H. rhamnoides* and other hosts, prior to the host shift. Feder et al. [59] found genetic divergence between apple and hawthorne host races of *Rhagoletis pomonella* L. pre-dating the introduction of the apple to North America. Given the long life history of *H. hippophaecolus* and brief planting history of *H. rhamnoides* in Jianping, we suppose the latter scenario is the case. Seabuckthorn is native to parts of western and northern China although records for the historical host plant use by *H. hippophaecolus* are lacking. Our results indicate that an *H. hippophaecolus* lineage might have adapted to utilize *H. rhamnoides* in China prior its spread. The possibilities of an ancestral host shift and stable host-associated genetic divergence in seabuckthorn carpenter moth are suggested.

We found no fixed diagnostic differences in AFLP data between the different host-associated forms. Host-associated genetic divergence should also be further demonstrated by sampling additional populations feeding on different host plants in more locations. In future studies, more different genetic markers are recommended in this system. They should include co-dominant markers such as microsatellites (not currently available for this species) and incorporation of variable regions of the mitochondrial genome. Microsatellites are highly polymorphic, locus-specific and can show co-dominant inheritance. They may recover higher levels of variability than other markers, particularly if following a population bottleneck associated with host shift. Mitochondrial sequences can be analyzed to determine patterns of evolutionary relationships between different haplotypes. This may provide information on the historical evolution of host-associated forms in the seabuckthorn moth.
